# The impact of the hypoxia‐VEGF‐vascular permeability on COVID‐19‐infected patients

**DOI:** 10.1002/EXP.20210051

**Published:** 2021-10-30

**Authors:** Yihai Cao

**Affiliations:** ^1^ Department of Microbiology, Tumor and Cell Biology Karolinska Institute Stockholm Sweden

**Keywords:** COVID‐19, therapy, VEGF

## Abstract

Effective treatment of patients with severe COVID‐19 to reduce mortality remains one of the most challenging medical issues in controlling unpredictable emergencies caused by the global pandemics. Unfortunately, such effective therapies are not available at this time of writing. In this article, I discuss the possibility of repurposing clinically available anti‐VEGF (vascular endothelial growth factor) drugs that are routinely used in oncology and ophthalmology areas for effective treatment of patients with severe and critical COVID‐19. Our preliminary findings from a clinical trial support the therapeutic concept of using anti‐VEGF for treating patients with severe COVID‐19 to reduce mortality. The aim of this article is to further provide mechanistic insights into the role of VEGF in causing pathological changes during COVID‐19 infection.

## Graphical abstract

Leaking plasma from blood vessels triggered by COVID‐19 infection plays a crucial role in causing pulmonary atelectasis (lung collapse), which fills up the lung air sacs with liquid and the lung becomes deflated. If sufficiently large areas of the lung tissue become deflated by this problem, the lung tissue can no longer take enough air and oxygen for the entire body and patients suffer from life‐threatening shortness of breath. This article discusses the possibility of repurposing clinically available drugs designed for treating cancer and eye diseases for the benefit of treating patients with severe COVID‐19 by blocking vascular leakage.

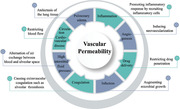

On March 11 of 2020, the World Health Organization (WHO) announced Coronavirus disease‐19 (COVID‐19) as a pandemic infectious disease that affected nearly all countries around the globe. At this time of writing of May 6, 2021, the total number of COVID‐19‐infected cases excesses 155,000,000 and 3,240,000 deaths and new cases continue rising in most countries.^[^ ^1^ ^]^ According to statistics on May 4, 2021, 617,721,160 people have received the 1 dose of COVID‐19 vaccine and 285,448,913 people become fully vaccinated.^[^ ^2^ ^]^ The uncontrollable situation of COVID‐19 virus poses unprecedented challenges for political decision makers, medical specialists, feasibility of medical facilities, economic supports, education, service sectors, and basic research. Although stringent protective procedures such as physical isolation are recognized as the most effective approach for prevention, different countries have implemented various policies to battle the COVID‐19 pandemic. Vaccination remains the most effective approach to control the pandemic situation around the globe, provided future mutant COVID‐19 viruses remain sensitive to the vaccine‐triggered neutralizing antibodies.

Similar to severe acute respiratory syndrome‐coronavirus (SARS‐CoV), COVID‐19 virus belongs to a positive‐sense single‐stranded ribonucleic acid (RNA) betacoronavirus.^[^ ^3^ ^]^ Respiratory droplets spread within short distances are the main transmission pathway between humans. Among the four structural proteins, that is, the S (spike), E (envelope), M (membrane), and N (nucleocapsid) proteins, the spike protein is responsible for attaching to and fusing with the membrane of a host cell through angiotensin converting enzyme 2 (ACE2) receptor.^[^ ^4^, ^5^ ^]^ The receptor‐binding domain (RBD) of S protein exhibits the most variable part of the COVID‐19 genome.^[^ ^5^, ^6^ ^]^ Mutations of critical amino acids in RBD may change ACE2 binding affinity and pathology in humans. ACE2 is widely expressed many cell types, including, pneumocytes, cardiovascular cells, macrophages, and T‐lymphocytes.^[^ ^7^ ^]^ Thus, COVID‐19 virus likely infects these ACE2^+^ cells. Owing to high ACE2 expression, pneumocytes are the primary targets for COVID‐19.

COVID‐19‐infected patients manifest a wide‐spectrum of symptoms, including asymptomatic infection, mild upper respiratory tract illness, and severe pneumonia with respiratory failure.^[^ ^8^, ^9^ ^]^ Most patients belong to normal and mild, and their mortality is lower than SARS‐CoV and middle east respiratory syndrome‐coronavirus (MERS‐CoV). Common symptoms on admission were fever and dry cough, followed by sputum production and fatigue. Lymphocytopenia was observed in 82.1% of patients.^[^ ^10^ ^]^ On admission, 50% of the patients presented ground‐glass shadow on chest CT.^[^ ^8^ ^]^ Comorbidities were present in nearly half of patients, with hypertension being the most common comorbidity, followed by diabetes and coronary heart disease.^[^ ^11^ ^]^ CT scans of the chest provide a diagnostic value even before clinic symptom develops. Typically, CT‐scan reveals bilateral multilobar ground‐glass opacificities with a peripheral, asymmetric, and posterior distribution.^[^ ^12^ ^]^ Subpleural dominance, crazy paving (lobular septal thickening with variable alveolar filling), and consolidation develop along the disease progression. Pathological features include pleurisy, pericarditis, lung consolidation, and pulmonary edema. Mild pneumonia shows pulmonary edema, pneumocyte hyperplasia, large atypical pneumocytes, interstitial inflammation with lymphocytic infiltration, and multinucleated giant cell formation.^[^ ^13^, ^14^ ^]^ At the advanced and severe pneumonia, diffuse alveolar damage (DAD) with diffuse alveolar exudates becomes apparent and diffuse DAD is responsible for the acute respiratory distress syndrome (ARDS) and severe hypoxemia was observed in this disease.^[^ ^14^ ^]^ At the healing phase, reorganization of exudates in alveolar cavities and pulmonary interstitial fibrosis occurs. These pathological features indicate COVID‐19 severe patients suffer from high‐degree of pulmonary hypoxia.

Development of effective drugs for treating patients with severe COVID‐19 and reducing mortality is of the utmost importance and among the most urgent tasks. Although development of effective vaccines would provide a curative approach for prevention and treatment, there are a few challenging issues for vaccine development, including: (1) Timeline. Although a significant population has received vaccine, the pace of vaccination is far behind the expected schedule. Given the fact that a majority of population has not been vaccinated in most countries, the COVID‐19 pandemic‐associated mortality continues to rise to new records in certain countries; (2) COVID‐19 RNA viruses are genetically unstable and mutations frequently occur, creating new strains of COVID‐19 virus. The mutated COVID‐19 viral strains may become resistant to vaccines. At this time of writing, several mutations and genetic variants of COVID‐19 have been identified, especially in the S‐protein‐RBD.^[^ ^15^ ^]^ Genetic variations pose a challenge for development of effective and stable COVID‐19 vaccines; (3) Authority approval. Before immunization of human populations, anti‐viral vaccines need to be fully assessed for therapeutic and preventive efficacies and toxicity tolerance; and (4) Defective immunity. A majority of COVID‐19 patients show defective immunity by manifesting lymphopenia with very low counts of lymphocytes in their body.^[^ ^8^ ^]^ If so, it would be very challenging to evoke the immune system by immunization.

In confronting vaccine challenges, current therapeutic strategies are focusing on treating critical patients with life‐threatening severe symptoms such as ARDS to reduce mortality. To this end, repurposing existing drugs with diverse principles to alleviate clinical symptoms has received great attention.^[^ ^16^, ^17^ ^]^ Numerous trials have been launched to treat COVID‐19 patients by targeting virus and host pathological processes.^[^ ^16^, ^17^ ^]^ We provide a few examples, including (1) Anti‐inflammatory drugs. Blocking COVID‐19‐triggered cytokine storms by antibodies neutralizing various cytokines such as IL‐1 (interleukin‐1), IFN (interferon)‐γ, IL‐6 (interleukin‐6), and granulocyte‐macrophage colony‐stimulating factor (GM‐CSF) is an undisputed approach for treating severe patients.^[^ ^16^, ^18^ ^]^ Several pharmaceutical companies and medical research centers are sponsoring these trials and results are inconclusive; (2) Hydroxyquinoline shows both prophylactic and therapeutic advantage displays by displaying potent antiviral effects on COVID‐19 infection.^[^ ^19^, ^20^ ^]^ Mechanistically, quinolone elevates endosomal pH and interferes with terminal glycosylation of the cellular receptor, angiotensin‐converting enzyme 2, which negatively influences the virus‐receptor binding and abrogates the infection; (3) Stem cell therapy. Although mechanistically unclear, a few stem cell‐based trials are ongoing for treating COVID‐19 infection;^[^ ^21^ ^]^ (4) ACE2 inhibitors. Soluble ACE2 and small molecules have been developed to interfere COVID‐19‐ACE2 interaction for cell entry;^[^ ^22^ ^]^ (5) Protease inhibitors. Ritonavir and ASC09 are two examples to block viral protease activity;^[^ ^23^ ^]^ (6) Targeting endocytic and autophagy pathways;^[^ ^20^ ^]^ and (7) Antiviral drug nucleotide analogs. Remdesivir is an adenosine analog that incorporates into nascent viral RNA chains and causes their pre‐mature termination;^[^ ^24^ ^]^ (8) Corticosteroids. In the Randomized Evaluation of COVID‐19 Therapy (RECOVERY) trial of a multicenter, randomized, open‐label trial in hospitalized patients with COVID‐19, corticosteroids showed that the mortality from COVID‐19 was lower among patients who were randomized to receive dexamethasone than among those who received the standard of care;^[^ ^25^ ^]^ (9) Antithrombotic therapy. Hospitalized non‐pregnant adults with COVID‐19 are recommended to receive a prophylactic dose of anticoagulation.^[^ ^26^ ^]^

Along COVID‐19 infection, patients often develop dyspnea, shortness of breath, owing to lung inflammation, plasma extravasation, deflated alveoli, and atelectasis. Dyspnea triggers local hypoxia in the lung tissue and systemic hypoxia in all tissues. Hypoxia induces a series of pathological responses by transcriptional activation of hypoxia‐inducible factor (HIF)^[^ ^27^ ^]^ (Figure 1). Vascular endothelial growth factor (VEGF) is one of the key HIF‐targeted genes and hypoxia is a potent driver for upregulation of VEGF expression.^[^ ^28^ ^]^ VEGF is a vascular factor that displays multiple biological functions under physiological and pathological conditions, including embryonic development, hematopoiesis, vasculogenesis, angiogenesis, vascular permeability, inflammation, neurogenesis, metabolism, endocrine regulation, tumor growth, cerebrocardiovascular disease, and ophthalmological disease.^[^ ^29^ ^]^ Among these known functions, angiogenesis and vascular permeability are probably the most well‐characterized vascular functions of VEGF. In addition to its angiogenic activity, VEGF potently induces vascular permeability through the VEGFR2 (VEGFR; vascular endothelial growth factor receptor) receptor‐mediated alterations of vascular fenestration and interendothelial junction.^[^ ^30^ ^]^ I propose that, in the COVID‐19‐infected lung tissue, high levels of VEGF instigates plasma extravasation, leading to pulmonary edema (Figure 1). The deflated alveoli filled with plasma serve as a nutritious matrix for microbial infection, including bacterial and fungal infections. The cytokine‐rich plasma augments lung inflammation by recruiting inflammatory cells such as macrophages and neutrophils. Additionally, the extravasated plasma coagulates to form a gel‐like matrix that blocks air exchange between the alveolar space and surrounding capillaries. Plasma clots also provide a matrix for invasion of angiogenic vessels, fibrotic cells, and other cellular structures. Ultimately, lung atelectasis aggravates dyspnea, hypoxia, acute respiratory distress syndrome, and development of the severe COVID‐19 syndrome. Therefore, the hypoxia‐induced vascular leakage plays a central role in causing a myriad of pathological responses during COVID‐19 infection (Figure 2).

**FIGURE 1:**
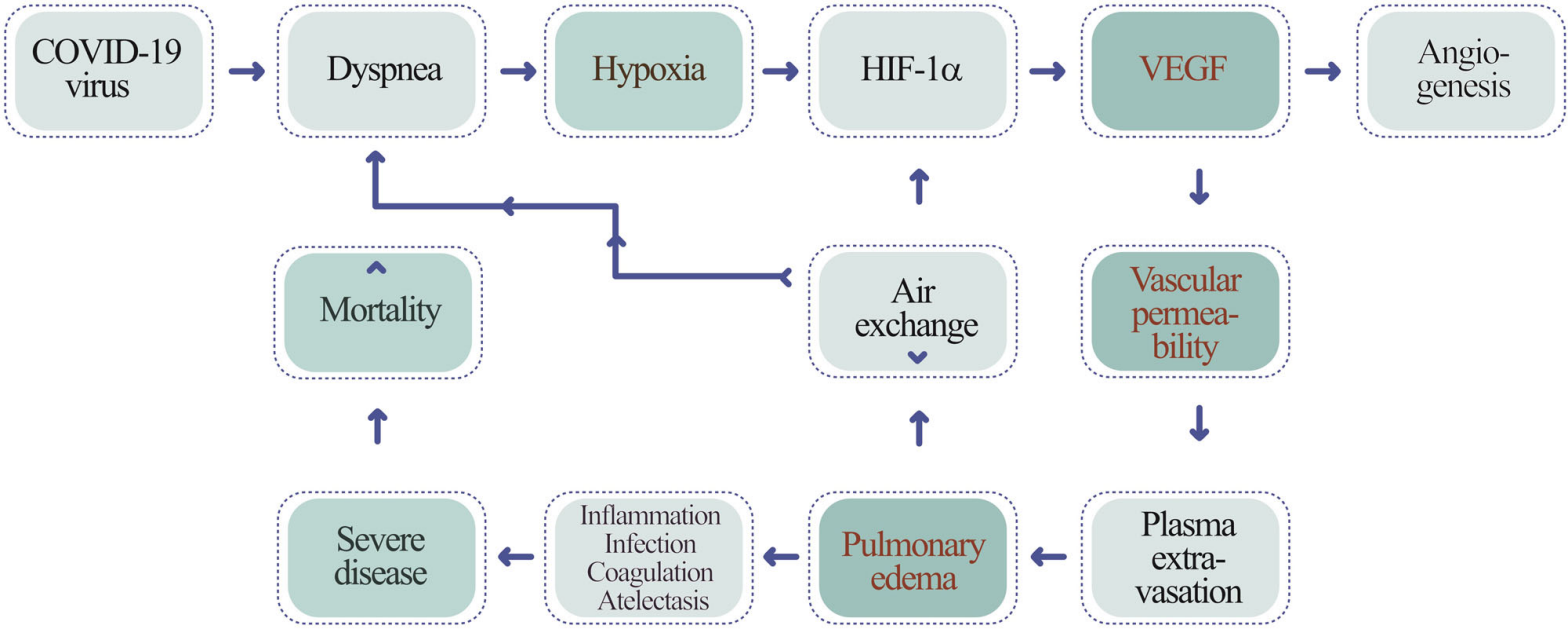
Mechanistic diagram explaining COVID‐19 infection‐triggered the hypoxia‐VEGF‐vascular permeability axis in causing severe disease and mortality. COVID‐19 infection induces expiratory dyspnea and ARDS, which create severe pulmonary hypoxia. Hypoxia induces VEGF expression through HIF‐1α and VEGF increases vascular leakage and plasma extravasation, leading to development of pulmonary edema. Pulmonary edema aggravates angiogenesis, lung inflammation, infection, alveolar coagulation, and atelectasis, development of severe clinical symptoms, and ultimately high mortality

**FIGURE 2:**
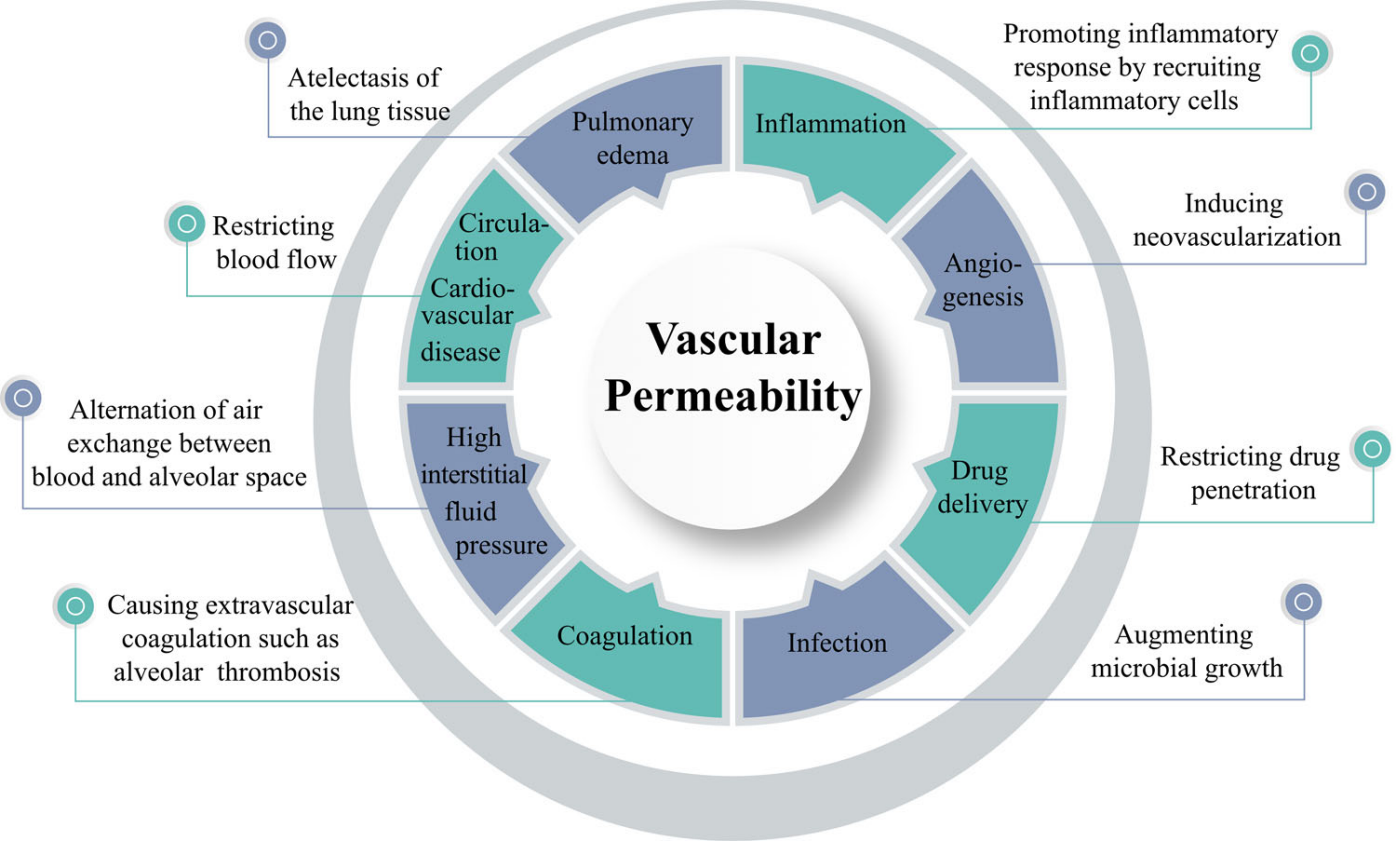
The hallmark of vascular permeability in development of clinical symptoms of COVID‐19. Vascular permeability plays a central role in causing pathological changes of the COVID‐19‐infected lung tissues and other tissues, including exacerbation of inflammation, angiogenesis, infection, extravascular coagulation, and pulmonary edema. The increase of interstitial fluid pressure restricts blood perfusion in capillaries and microvessels, which further exacerbates tissue hypoxia and dyspnea. Therefore, targeting vascular permeability provides an imperative approach for treating patients with severe COVID‐19. This therapeutic concept can be extended to other infectious and non‐infectious diseases in which vascular degeneration and leakage participate in pathological alterations

It is worth mentioning that the COVID‐19‐infected lung tissue somehow resembles that of solid tumors, which also contain disorganized and high‐permeable microvessels.^[^ ^31^ ^]^ Owing to the leaky feature of tumor vessels, the interstitial fluid pressure (IFP) is considerably high within the tumor tissue.^[^ ^32^ ^]^ In theory, high IFP would press on capillaries which often lack supportive perivascular cells to restrict blood flow. If so, drug delivery will be impeded, resulting insufficient penetration of drugs into the tissues. Although this hypothesis has not been tested in the COVID‐19‐infected lung tissue and other tissues, it warrants further investigation and clinical validation.

On the basis of these mechanisms, targeting the VEGF‐VEGFR signaling would provide a valid approach for treating patients with severe COVID‐19. From February to April of 2020, we conducted a 2‐center clinical trial by recruiting 26 patients with severe COVID‐19 to receive treatment with a single dose of 7.5 mg/kg bevacizumab, an anti‐VEGF neutralizing antibody.^[^ ^33^ ^]^ The recruitment criteria include partial arterial oxygen pressure to fraction of inspiration O_2_ ratio (PaO_2_/FiO_2_) > 100 mmHg and ≤300 mmHg and diffuse pneumonia confirmed by chest imaging. By comparison with standard care alone, bevacizumab plus standard care improves the PaO_2_/FiO_2_ ratio after 24 h. By day 28, 92% patients demonstrate oxygen‐support improvement, 65% patients are discharged, and none show worsen oxygen support or death. In addition to improvement of oxygen support, 93% of bevacizumab‐treated patients rapid abatement of fever within 72 h, including those with high and persistent fever. Other clinical parameters, including lymphopenia and inflammation are also significantly improved in the bevacizumab‐treated patients compared with controls. Although improvement of pulmonary functions by anti‐VEGF therapy has been emphasized in this trial, functional improvements of other affected tissues such as liver, kidney, heart, and immune response likely provide additional mechanisms for clinical benefits as these tissues and organs also participate in COVID‐19‐triggered pathological responses.^[^ ^34^ ^]^

These clinical data support the fact that VEGF‐induced vascular changes play a crucial role in causing life‐threatening defects of clinical symptoms and blocking the VEGF signaling provides an important approach for treating patients with severe COVID‐19. These clinical findings are validated by an independent study.^[^ ^35^ ^]^ Although these small single‐arm trials, these clinical data show promises of the anti‐VEGF and anti‐VEGFR in clinical management of COVID‐19‐infected patients. If these findings are further validated by randomized controlled trial in large cohort studies, this anti‐VEGF‐based therapeutic approach would likely become an important therapeutic component for treating COVID‐19 patients.

## CONFLICT OF INTEREST

Yihai Cao is a member of the *Exploration* editorial board. The author claims no conflict of interests.
